# Editorial: Ultrasound in rheumatology—A polyhedric imaging tool

**DOI:** 10.3389/fmed.2023.1150111

**Published:** 2023-02-07

**Authors:** Andrea Di Matteo, Christian Dejaco

**Affiliations:** ^1^Rheumatology Unit, Department of Clinical and Molecular Sciences, Polytechnic University of Marche, “Carlo Urbani” Hospital, Ancona, Italy; ^2^Faculty of Medicine and Health, Leeds Institute of Rheumatic and Musculoskeletal Medicine, University of Leeds, Leeds, United Kingdom; ^3^Department of Rheumatology, Medical University Graz, Graz, Austria; ^4^Department of Rheumatology, Hospital of Bruneck (ASAA-SABES), Teaching Hospital of the Paracelsus Medical University, Bruneck, Italy

**Keywords:** ultrasound, rheumatology, joint, soft tissues, imaging

In recent years, ultrasound (US) has become a pivotal tool in the diagnosis, differential diagnosis, follow-up and management of patients with rheumatic and musculoskeletal diseases (RMDs), such as rheumatoid arthritis (RA), spondylarthritis (SpA), crystal arthropathies and connective tissue diseases (CTDs) (1–4). Recent studies have also highlighted the potential value of this technique in the assessment of extra-articular “targets” of rheumatic diseases, such as muscles, salivary glands, nerves, skin and lungs. In large vessel vasculitis, the key diagnostic role of US has widely been recognized (5).

The articles included in the current Research Topic bring new insights into the application of US in the diagnosis and characterization of joint and extra-articular manifestations in a broad spectrum of RMDs. Although focused on US, research on other imaging techniques have been presented, as well as a comparison between US and other imaging methodologies, which is important for the understanding of the role of US in the assessment of RMDs.

Previous studies have shown the promising role of US in improving the prediction for the development of RA in individuals “at-risk” for this disease (6). In the current Research Topic, Harnden et al. reviewed the value of available imaging modalities for this purpose, including US, but also conventional radiography, magnetic resonance imaging (MRI), quantitative computed tomography (CT) and nuclear imaging, and the rationale for their use in the main populations “at-risk” of RA. According to the algorithm proposed for the use of imaging in these “at-risk” populations, US should be used as first line in “at-risk” individuals with musculoskeletal (MSK) symptoms (i.e., clinically suspect arthralgia or ACPA positive individuals with inflammatory MSK symptoms). If the US is negative, MRI may be used to identify sub-clinical inflammation, particularly at extracapsular structures. Radiographs should be used in “at-risk” individuals with non-inflammatory MSK symptoms to rule out alterative diagnoses. In “at-risk” individuals with no MSK symptoms (i.e., asymptomatic first-degree relatives or ACPA positive individuals), no imaging may be required given the absence of evidence suggesting the diagnostic or predictive value of any imaging techniques in this population.

Carbonell-Bobadilla et al. explored whether RA patients show a different phenotype according to the presence or absence of RA-related autoantibodies. The authors found that seronegative RA patients (i.e., negative for ACPA and rheumatoid factor) had a later disease onset and required less anti-rheumatic therapies than seropositive patients. In contrast, seropositive RA patients revealed more inflammation and joint damage on US.

In a study on human cadavers, Baksa et al. provided a high-resolution anatomical map on the arterial vasculature of healthy human metacarpophalangeal joints, thus providing an important basis for the evaluation of the vascularization of these joints by US.

Ventura-Rios et al. compared the extent of enthesitic changes in patients with SpA and gout using the US-based Madrid Sonographic Enthesis Index (MASEI). The overall MASEI was similar in both groups, however the prevalence and distribution of the US findings indicating inflammation and structural damage varied depending on the enthesis evaluated. While the prevalence of bone erosions and power Doppler signal was higher in patients with SpA in comparison with gout (especially at the Achilles tendon enthesis), gout patients had a higher prevalence of US structural damage, including calcifications/enthesophytes, especially at the proximal patellar tendon.

Geng et al. investigated the value of US detected tenosynovitis and/or enthesitis in improving the diagnosis of PsA according to the CASPAR criteria, using the clinically based diagnosis by the rheumatologist as the gold standard. In this study, 326 consecutive patients [164 with PsA and 162 with other conditions, such as psoriasis (PsO), osteoarthritis with PsO/family history of PsO, fibromyalgia with PsO, seronegative RA and undifferentiated arthritis] were consecutively enrolled. The diagnostic accuracy for CASPAR criteria increased from 89.3% to 93.6% when US was added to the clinical assessment in the included patients.

In a systematic literature review (SLR), Sakellariou et al. analyzed the evidence on the applications of US for the detection of subclinical involvement of joints and entheses in patients with inflammatory bowel diseases (IBD) with no previous history of inflammatory arthritis (“non-arthropathic IBD”). The main finding of this SLR was the high variability in the frequency of both chronic and acute inflammatory US lesions in patients with IBD, particularly in regard to the overall prevalence of the joint and entheseal abnormalities (mainly power Doppler signal). The limited evidence supporting the use of US in this population highlighted the need for more research on this topic.

Temporomandibular joint (TMJ) disorders are common in the general population as well as in patients with rheumatic diseases. In a narrative review, Maranini et al. discussed the role of US and MRI in the evaluation of TMJ disorders, proposing an imaging-based algorithm for the diagnosis of these conditions. According to the authors, US should be used as an “entry-criterion,” given the high availability and relatively low costs of this imaging technique, including asymptomatic “at-risk” patients, such as those with RA. MRI (or CT scan in alternative) could be used in case of a non-conclusive US examination as well as during follow-up.

Barbosa-Cobos et al. developed a novel technique using pixel analysis (i.e., image J) for the interpretation of sono-elastography in the assessment major salivary glands. This methodology helped to discriminate between patients with Sjogren's syndrome and healthy subjects and revealed a significant association with patients' clinical features (i.e., “sicca symptoms”), objective evaluation of ocular and oral dryness, and histologic findings.

In a web-based multicentric study involving 42 rheumatologists and 2 radiologists from 13 countries, Di Matteo et al. highlighted the good inter and intra-rater reliability of two recently developed visual scales for the assessment of US muscle echogenicity using images and clips from patients with different rheumatic diseases. The scope of these scales is to detect (the often underestimated) sarcopenia “early” in patients with RMD (7).

Two articles focused on the role of US in giant cell arteritis (GCA). In a narrative review, Kirby et al. discussed the current use of US in routine clinical practice in patients with GCA, as well as the current evidence on the reliability and applicability of this imaging technique, highlighting the importance of incorporating US into diagnostic algorithms to improve the diagnosis of GCA. In a retrospective study, López-Gloria et al. developed new cut-off values for the measurement of intima-media thickness of cranial and extra-cranial arteries to discriminate between patients with and without GCA. These newly proposed cut-off values could potentially improve the diagnostic accuracy of US in this condition.

Finally, Zaottini et al. provided an overview on the role of US in the assessment of peripheral neuropathies of rheumatological interest, describing the different pathologic features and patterns of nerve involvement observed in the most common RMDs, such as Sjogren's syndrome, systemic sclerosis, RA, PsA, Behcet's syndrome, small and large vessels vasculitis, sarcoidosis and crystal arthropathies. The authors also described the potential advantages and limitations of using US for the assessment of peripheral neuropathies in comparison with other imaging techniques, such as MRI, and in addition to the diagnostic work-up that is routinely carried out in these conditions (e.g., nerve conduction study).

In conclusion, the current Research Topic provides a collection of papers, which highlights the recent advances, and potential applications of US in several RMDs. Several articles also showed the potential value of US in the assessment of extra-articular manifestations in patients with CTDs and large vessel vasculitis.

## Author contributions

ADM and CD are guest editors of this Research Topic. All authors have made a substantial, direct, and intellectual contribution to the work and approved it for publication.
